# Conservative Treatment of Anteriorly Angulated Post-traumatic Coccydynia: A Case Report

**DOI:** 10.7759/cureus.106466

**Published:** 2026-04-05

**Authors:** Sarra El-Hamlili, Amal Aoussaf, Mohamed Harmouche, Siham Elmir

**Affiliations:** 1 Physical and Rehabilitation Medicine, Mohammed VI University Hospital Center, Oujda, MAR; 2 Faculty of Medicine and Pharmacy, Mohammed Ist University, Oujda, MAR

**Keywords:** anteriorly angulated coccyx, conservative treatment, pain management, post-traumatic coccydynia, rehabilitation

## Abstract

Coccydynia refers to pain in the coccygeal region, typically exacerbated by sitting and commonly resulting from direct trauma or difficult childbirth. We report the case of a 34-year-old woman presenting with persistent coccygeal pain one month after falling down a flight of stairs. Clinical examination revealed localized tenderness over the coccyx, aggravated by sitting, while lateral radiography demonstrated anterior angulation of the coccyx. Conservative treatment was initiated, including avoidance of prolonged sitting, use of a coccygeal cushion, local analgesic therapy, and a structured physiotherapy program. At three months, the patient’s pain decreased from 7/10 to 2/10 on the VAS, with improved sitting tolerance. This case highlights the effectiveness of individualized conservative treatment in post-traumatic coccydynia before any invasive interventions.

## Introduction

Coccydynia is a painful condition affecting the coccyx, often underdiagnosed, and can significantly impair patients’ quality of life, especially during prolonged sitting. It affects women approximately four times more frequently than men [1]. Its etiology is multifactorial, including traumatic events such as falls onto the buttocks [2] or difficult vaginal deliveries, anatomical variations of the coccyx, and hypermobility of coccygeal segments. These factors may lead to morphological abnormalities, such as anterior angulation, which, although relatively rare, can significantly contribute to the development of coccydynia. Diagnosis is often challenging due to the possibility of referred pain from the lumbosacral spine or sacroiliac joints and the lack of specific clinical or radiological criteria in some cases.

Initial management relies on a multidisciplinary conservative approach, including lifestyle modifications, analgesic therapy, and a structured rehabilitation program. However, in cases of persistent symptoms, other therapeutic options may be considered, such as local corticosteroid injections, pulsed radiofrequency, or, as a last resort, surgical intervention [3].

This case report highlights the importance of early diagnosis and a combined therapeutic approach in the management of post-traumatic coccydynia, particularly in the presence of anterior coccygeal angulation.

## Case presentation

We report the case of a 34-year-old female patient, a teacher, with no prior history of coccydynia, who had undergone two cesarean deliveries. She presented to the Department of Physical Medicine and Rehabilitation with persistent coccygeal pain that developed one month after a fall onto the buttocks.

The pain was mechanical in nature, localized to the coccyx, non-radiating, and aggravated by prolonged sitting and positional changes. It was resistant to previous analgesic treatment, as the patient had received a nonsteroidal anti-inflammatory drug (NSAID) at a dose of 50 mg, taken twice daily for seven days, without significant relief. Pain intensity was rated at 7/10 on the visual analog Scale (VAS).

Clinical examination revealed localized tenderness on palpation of the coccygeal tip, reproducing the patient’s pain. Examination of the lumbar spine and sacroiliac joints, including specific provocation tests, was unremarkable, supporting the diagnosis of isolated post-traumatic coccydynia. Neurological examination of the lower limbs was normal, with no motor or sensory deficits.

**Figure 1:**
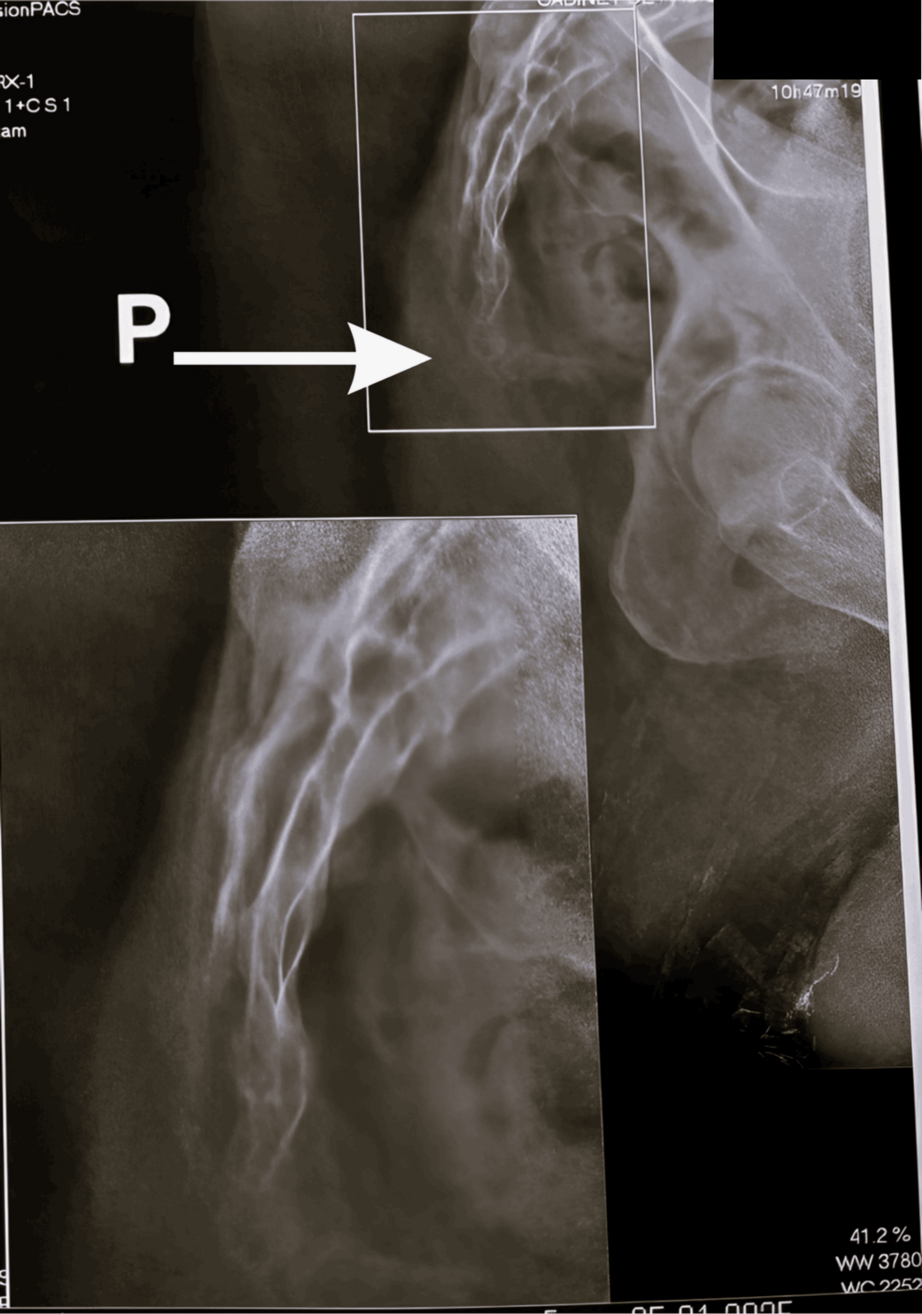
Lateral pelvic radiograph showing anterior angulation of the coccyx The radiograph demonstrates anterior angulation of the coccyx (white arrow), consistent with the patient’s post-traumatic coccydynia.

A lateral pelvic radiograph demonstrated anterior angulation of the coccyx (Figure 1), consistent with the clinical presentation. Based on the clinical and radiological findings, a diagnosis of post-traumatic coccydynia associated with anterior coccygeal angulation was established.

Management was based on a structured conservative approach, including activity modification to avoid prolonged sitting and rapid sit-to-stand transitions, as well as the use of a U-shaped coccygeal cushion to reduce local pressure.

The rehabilitation program, conducted three times per week, included analgesic physiotherapy with transcutaneous electrical nerve stimulation (TENS) for 20 minutes per session. It also comprised strengthening exercises targeting the pelvic floor, gluteal, and abdominal muscles (10-15 repetitions per set), stretching of the hip flexors, internal stretching of the levator ani muscle, and mobilization of the lumbosacral junction for 10 minutes per session.

A personalized home exercise program was prescribed to maintain therapeutic gains and improve tolerance to daily activities. Postural and ergonomic advice for the workplace was also provided. The patient was reassessed every 10 sessions to monitor pain progression and adjust the rehabilitation program accordingly.

At the three-month follow-up, the patient reported significant improvement, with pain decreasing from 7/10 to 2/10 on the visual analog scale (VAS), along with enhanced sitting tolerance and overall functional capacity.

## Discussion

Coccydynia is defined as pain localized to the coccyx, typically exacerbated by prolonged sitting or during the transition from sitting to standing, which corresponds to the symptoms reported by our patient. It occurs more frequently in women, which has been attributed to anatomical, hormonal, and biomechanical differences in the female pelvis [4]. Among the most common etiologies are repetitive microtrauma, difficult childbirth [5,6], and direct trauma to the coccyx [7], such as falls onto the buttocks or impact during sports activities, all of which can lead to persistent coccygeal pain. These traumatic events may result in structural changes, such as anterior angulation of the coccyx, leading to mechanical pain in the sitting position.

In our patient, the onset of coccydynia was attributed to an acute traumatic event, namely, a fall on the stairs, which occurred one month prior to consultation. She had no history of difficult childbirth, as both deliveries were performed by cesarean section. This absence of obstetric strain further supports the hypothesis that the anterior angulation of the coccyx resulted from direct trauma.

The diagnosis of coccydynia is primarily based on careful clinical evaluation. Patient history typically reveals localized coccygeal pain exacerbated by prolonged sitting or when transitioning to a standing position. Physical examination includes direct palpation of the coccyx, which often reproduces the pain and is considered a characteristic finding. Rectal examination may also help assess coccygeal mobility and segmental tenderness, particularly when external findings are inconclusive.

Although the diagnosis is mainly clinical, imaging plays an important role in confirming or clarifying the anatomical origin of pain, especially in cases of persistent symptoms or when non-conservative treatment is being considered [8]. Standard lateral radiographs can identify structural abnormalities, such as abnormal angulation, dislocation, or bony spicules, which may explain the pain. Dynamic radiographs, comparing sitting and standing positions, can further assess coccygeal mobility and detect instability or subluxation not visible on static images [9].

In our case, anterior angulation of the coccyx was clearly demonstrated on the lateral radiograph, consistent with the clinical findings. Recent radiological studies have shown that anatomical variations of the coccyx, including increased ventral curvature and other morphological abnormalities, are common in patients with coccydynia and may contribute to unfavorable biomechanical dynamics, explaining mechanical pain during weight-bearing in the sitting position and postural transitions [10].

Initial management is based on conservative measures, which are often effective, particularly in recent post-traumatic cases before chronicity develops. Systematic reviews have shown that these approaches can reduce pain and improve function without the need for invasive intervention. Physiotherapy interventions, including joint mobilization, manual therapy, muscle stretching, and therapeutic exercises, have been associated with significant symptom improvement [5,11].

In our patient, the therapeutic strategy was strictly conservative. It included avoidance of prolonged sitting and the use of a pressure-relieving cushion to reduce coccygeal load, along with local analgesic therapy to relieve symptoms and improve daily comfort. In parallel, a structured functional rehabilitation program was implemented. The overall objective was to reduce pain, improve coccygeal mobility, and restore muscular and postural function, thereby enhancing daily comfort.

This approach is consistent with findings from systematic reviews supporting the combined use of ergonomic measures, postural adjustments, and individualized physiotherapy programs as first-line conservative treatment. The favorable outcome observed in our patient, with a reduction in pain from 7/10 to 2/10 at three months and improved tolerance to sitting, illustrates the potential effectiveness of a well-structured conservative approach [5].

However, some patients may require, after failure of strict conservative management, advanced non-surgical interventions such as local corticosteroid or anesthetic injections aimed at reducing inflammation and pain. These interventions are generally reserved for patients with persistent symptoms despite appropriate conservative treatment and significant impairment in quality of life. Clinical outcomes vary, likely due to heterogeneity in treatment protocols across studies [12].

As a last resort, surgical interventions, such as coccygectomy, may be considered, with careful evaluation of risks and benefits based on patient characteristics and response to prior treatments [13].

## Conclusions

Post-traumatic coccydynia will remain a common cause of coccygeal pain and may result from falls or other direct trauma. This case illustrates that anterior angulation of the coccyx, if identified radiologically, can be associated with significant mechanical pain even in the absence of difficult childbirth or other obstetric risk factors. Conservative management, combining postural adaptations, local analgesic treatment, and targeted functional rehabilitation, is expected to lead to a marked improvement in symptoms. This case highlights the importance of a comprehensive clinical evaluation, complemented by appropriate imaging, to guide therapeutic decision-making and to limit the need for early invasive interventions.

It should be noted that these findings are based on a single case, and future studies will be required to confirm the generalizability of these observations. Nevertheless, this case emphasizes that early diagnosis and an individualized conservative approach will optimize outcomes, relieve pain, restore function, and help avoid potentially unnecessary surgical interventions.
